# *MEIS1* knockdown may promote differentiation of esophageal squamous carcinoma cell line KYSE‐30

**DOI:** 10.1002/mgg3.746

**Published:** 2019-05-14

**Authors:** Reihaneh Alsadat Mahmoudian, Bahareh Bahadori, Abolfazl Rad, Mohammad Reza Abbaszadegan, Mohammad Mahdi Forghanifard

**Affiliations:** ^1^ Immunology Research Center Mashhad University of Medical Sciences Mashhad Iran; ^2^ Department of Biology, Damghan Branch Islamic Azad University Damghan Iran; ^3^ Cellular and Molecular Research center Sabzevar Univeristy of Medical Sciences Sabzevar Iran; ^4^ Medical Genetics Research Center Mashhad University of Medical Sciences Mashhad Iran; ^5^ Department of Biology, Faculty of Science Ferdowsi University of Mashhad Mashhad Iran

**Keywords:** differentiation, ESCC, KYSE‐30, *MEIS1*, *TWIST1*

## Abstract

**Background:**

*MEIS1* (Myeloid ecotropic viral integration site 1), as a homeobox (HOX) transcription factor, has a dual function in different types of cancer. Although numerous roles are proposed for *MEIS1* in differentiation, stem cell function, gastrointestinal development and tumorigenesis, the involved molecular mechanisms are poor understood. Our aim in this study was to elucidate the functional correlation between *MEIS1*, as regulator of differentiation process, and the involved genes in cell differentiation in human esophageal squamous carcinoma (ESC) cell line KYSE‐30.

**Methods:**

The KYSE‐30 cells were transduced using recombinant retroviral particles containing specific shRNA sequence against *MEIS1* to knockdown *MEIS1* gene expression. Following RNA extraction and cDNA synthesis, mRNA expression of *MEIS1* and the selected genes including *TWIST1, EGF, CDX2,* and *KRT4* was examined using relative comparative real‐time PCR.

**Results:**

Retroviral transduction caused a significant underexpression of *MEIS1* in GFP‐hMEIS1 compared to control GFP cells approximately 5.5‐fold. While knockdown of *MEIS1* expression caused a significant decrease in *EGF* and *TWIST1* mRNA expression, nearly ‐8‐ and ‐12‐fold respectively, it caused a significant increase in mRNA expression of differentiation markers including *KRT4* and *CDX2*, approximately 34‐ and 1.14‐fold, correspondingly.

**Conclusion:**

*MEIS1* gene silencing in KYSE‐30 cells increased expression of epithelial markers and decreased expression of epithelial‐mesenchymal transition (EMT) marker *TWIST1*. It may highlight the role of *MEIS1* in differentiation process of KYSE‐30 cells. These results may confirm that *MEIS1* silencing promotes differentiation and decreases EMT capability of ESC cell line KYSE‐30.

**Abbreviations**
ALL: Acute lymphoblastic leukemias
bHLH: Basic helix‐loop‐helix
BMP: Bone morphogenetic protein
ccRCC: Clear cell renal cell carcinoma
CSC: Cancer stem cell
EC: Esophageal cancer
EGF: Epidermal growth factor
ESC: Esophageal squamous carcinoma
ESCC: Esophageal squamous cell carcinoma
GC: Gastric cancer
HSC: Hematopoietic stem cell
ID: Inhibition of differentiation
MEIS1: Myeloid ecotropic viral integration site 1
MPNST: Malignant peripheral nerve sheath tumor
MSC: Mesenchymal stem cell
NSCLC: Non‐small‐cell lung cancer
SC: Stem cell
TALE: Three amino acid loop extension
TF: Transcriptional factor

## 1 INTRODUCTION

Malignancy is a complex heterogeneous illness introduced through accumulation of different damaging genetic and epigenetic alterations in tumor cells. Notably, disruption of various signaling networks and multiple molecular mechanisms involved in tumor onset and development can lead to extensive deregulation of gene expression profiles in human cancers (Beerenwinkel, Schwarz, Gerstung, & Markowetz, 2014; Chatterjee et al., 2018; Du & Che, 2017). Among identified genetic changes in the cancer etiology, abnormal expression of different gene categories such as tumor suppressors, oncogenes, DNA repair genes, stem cell‐related surface markers and cancer stem cells (CSCs) specific transcriptional factors (TFs), can be noted as leading cause of tumorigenesis (Sadikovic, Al‐Romaih, Squire, & Zielenska, 2008; Zhao, Li, & Zhang, 2017).

Based on experimental and theoretical data, there are associations between the expression of CSCs markers and cancer‐related genes in many tumors. CSCs preserve self‐renewal and proliferative potential via inhibiting differentiation signaling pathways during cancer initiation and development (Jin, Jin, & Kim, 2017; Lathia & Liu, 2017). Remarkably, the balance between differentiation and self‐renewal capabilities of CSCs produces the bulk of heterogeneous tumor mass contributing in aggressive and stemness phenotypes (Lathia & Liu, 2017). Specific gene expression profiles are needed for tumor cell differentiation which are dictated through different signaling pathways, transcription factor activities, as well as epigenetic alterations such as DNA modifications (Jögi, Vaapil, Johansson, & Påhlman, 2012). The involved signaling pathways in CSCs differentiation are BMP (bone morphogenetic protein) and RA (retinoic acid) pathways, while CSCs stemness signaling cascades include JAK/STAT, Wnt/β‐catenin, Hedgehog, Notch, PI3K/PTEN, and NF‐ kB (Jin et al., 2017; Matsui, 2016). The expression profile of differentiation‐associated genes is heterogeneous in nearly all types of tumor cells, probably due to the transcriptional activity of a small population of CSCs in combination with numerous partially differentiated cells (Palmer, Schmid, Berger, & Kohane, 2012). Inhibition of differentiation happens through highly expressed ID (Inhibitor of DNA‐binding/ differentiation) proteins as regulators of cell fate (Jin et al., 2017).

The most important targets for ID family are basic helix‐loop‐helix (bHLH) transcription regulators, and homeobox genes encoding DNA‐binding domain proteins (Jin et al., 2017; O'Toole et al., 2003).

HOX genes family members, as a subset of homeobox genes, encode TFs with fundamental roles in embryo development and segmentation, as well as differentiation of stem cell (Crist, Roth, Waldman, & Buchberg, 2011; Seifert, Werheid, Knapp, & Tobiasch, 2015). Abnormal expression of HOX genes, often accompanied by DNA hypermethylation, can lead to the developmental diseases and carcinogenesis. The transcribed TFs from HOX genes present two homeodomain groups consisting of a conserved 60 amino acids for sequence‐specific binding to DNA motifs and a three amino acid loop extension (TALE) (Tsumagari et al., 2013).

*MEIS1* (myeloid ecotropic viral integration site 1, OMIM: 601739), as an activator for the HOX members, forms heterodimer complex with HOX transcription factors to recruit either transcriptional co‐activator or co‐repressor in a DNA sequence‐dependent manner, modulating expression of target genes. Numerous TFs including *PREP1, HOXA7, HOXA9,* and *CREB1* regulate *MEIS1* expression in different normal tissues and several tumor cells (Torres‐Flores, 2013). *MEIS1* has an essential role in regulation of stemness state of stem cells, transcription adjustment of self‐renewal genes, as well as involved genes in cell development and differentiation, playing an oncogenic role in several tumors (Dardaei et al., 2015; Rad et al., 2016). mRNA and protein expression of *MEIS1*, as well as its cofactors, were demonstrated in numerous types of malignancies such as leukemia, neuroblastoma, ovarian, renal cell carcinoma, pancreatic, colorectal, gastric, skin, and lung cancers, as well as malignant peripheral nerve sheath tumors (Aksoz, Turan, Albayrak, & Kocabas, 2018). In addition, it has been recently reported that *MEIS1* may have cancer stemness property in esophageal squamous cell carcinoma (ESCC) where its downregulation was inversely correlated with stage of progression and metastasis of the tumor (Rad et al., 2016).

Differentiation outcome in squamous epithelium of esophageal needs a serial activity of different specific differentiation‐associated genes, and any disruption in this chain may block differentiation process leading to squamous epithelial neoplasia, although the involved molecular mechanisms remain poorly understood (Luo et al., 2014).

Therefore, in the current study, we aimed to assess the impact of *MEIS1* gene knockdown on the expression pattern of differentiation‐associated genes including *TWIST1* (twist family bHLH transcription factor 1, OMIM: 601622), *EGF* (epidermal growth factor, OMIM: 131530), *KRT4* (Keratin 4, OMIM: 123940), and *CDX2* (caudal type homeobox 2, OMIM: 600297) in human ESC cell line KYSE‐30, to define probable linkage between *MEIS1* and differentiation state of the cells.

## 2 MATERIALS AND METHODS

### 2.1 Cell lines and culture condition

Human ESCC (KYSE‐30) and embryonic kidney (HEK293T) cell lines were purchased from the Pasteur Institute Cell Bank of Iran (http://en.pasteur.ac.ir/) and grown in RPMI 1640 medium (Biosera) and Dulbecco's modified Eagle's medium (DMEM; Biosera), respectively. Both culture media were supplemented with 10% heat‐inactivated fetal bovine serum (FBS; Gibco, USA), 100 U/ml, and 100 μg/ml penicillin‐streptomycin (Gibco, USA) at a humidified atmosphere 37°C with 5% CO_2_.

### 2.2 *MEIS1* gene expression knockdown

The lentiviral pLKO.1‐puro plasmid (Cat. No. SHC003) as a shRNA expression vector was obtained from Sigma‐Aldrich (St. Louis, MO). The pLKO.1‐puro plasmid DNA was consisted the green fluorescent protein (GFP) gene under the control of the cytomegalovirus (CMV) promoter which express shRNA construct targeting the human *MEIS1* (GenBank reference sequence: NM_002398.3). The psPAX2 as a packaging vector and the pMD2.G as a vector encoding the VSV‐G (G‐protein of the vesicular stomatitis virus) were achieved from Addgene (plasmids 12260 and 12259, respectively, Cambridge, MA). Twenty‐one micrograms of pLKO.1‐MEIS1 or 21 μg PCDH513b plasmid along with 21 μg of psPAX2 and 10 μg of pMD2.G were transiently cotransfected into HEK293T cells according to the standard calcium phosphate method for producing lentiviral particles. The supernatant containing viral particles was collected at 24 and 48 hr after transfection and filtered through 0.45‐μm filter (Orange, Belgium). Then, the supernatant was pelleted using ultracentrifugation (Beckman‐Coulter ultracentrifuge XL‐100K, USA) at 70,000 × g, 4°C for 1 hr and resuspended in fresh medium. For transduction of KYSE‐30 cells, cells were cultured at a density of 1 × 10^5 ^cells in 6‐well plates the day before transduction. On the day of infection, the culture media were replaced with fresh ones containing the lentiviruses for an additional 4–5 days. In order to select the infected cells, the transduced cells were treated with 2 µg/ml puromycin (Invitrogen Corporation, Carlsbad, CA). The transduced KYSE‐30 cells with recombinant lentiviral particles of GFP (control) and GFP‐shMESI1 were assayed using inverted fluorescence microscopy.

### 2.3 RNA extraction, cDNA synthesis, comparative real time PCR, and statistical analysis

Total RNA was isolated from GFP and GFP‐shMESI1 transduced ESCC cell line using Tripure reagent (Roche, Nutley, NJ), subsequently DNase I (Thermo Fisher Scientific, Waltham, MA) treatment was performed for preventing DNA contamination. The first strand complementary DNA (cDNA) synthesis was carried out by the oligo‐dT method according to the constructer's procedures (Fermentas, Lithuania). *MEIS1* mRNA knockdown was assessed using qRT‐PCR. Furthermore, relative comparative changes of *KRT4* (GenBank reference sequence: NM_002272.4), *CDX2* (GenBank reference sequence: NM_001265.5), *EGF* (GenBank reference sequence: NM_001963.5), and *TWIST1* (GenBank reference sequence: NM_000474.4) mRNA expression were assessed in *MEIS1* silenced compared to GFP control cells using a quantitative real‐time PCR (SYBR Green, AMPLIQON, Denmark) using gene‐specific primer sequences shown in Table 1 on a LightCycler^®^ 96 Real‐Time PCR System thermocycler (Roche, Germany). Glyceraldehyde 3‐phosphate dehydrogenase (*GAPDH*) housekeeping gene was used to normalize data. The 2^‐ΔΔCt^ method was applied to measure fold changes of gene expression (Forghanifard, Khales, et al., 2017; Rad et al., 2016). The test was performed triplicate and the thermal profile for *MEIS1, KRT4, CDX2, EGF,* and *TWIST1* included an initial denaturation at 95°C for 10 min, followed by 45 cycles 94°C (30 s), specific annealing temperature (30 s), and 72°C (30 s).

**Table 1:** Primer sequences used in real‐time PCR

Gene	Forward primer	Reverse primer	Annealing T, °C
MEIS1	ATGACACGGCATCTACTCGTTC	TGTCCAAGCCATCACCTTGCT	62
KRT4	GCCGTGAGCATCTCTGTG	TCCTCTATCGTCTCTTGTTCAG	58
CDX2	ACAGTCGCTACATCACCATC	GATTTTCCTCTCCTTTGCTC	55
EGF	ATGTAGCGGTTGTTCCTC	ATGGTTGTGGTCCTGAAG	54
TWIST1	GGAGTCCGCAGTCTTACGAG	TCTGGAGGACCTGGTAGAGG	57
GAPDH	GGAAGGTGAAGGTCGGAGTCA	GTCATTGATGGCAACAATATCCACT	60

Note

GenBank reference sequence for the examined mRNA: *MEIS*: NM_002398.3. *KRT4*: NM_002272.4, *CDX2*: NM_001265.5, *EGF*: NM_001963.5, and *TWIST1*: NM_000474.4.

The SPSS 19.9 statistical package (SPSS, Chicago, IL, USA) was applied for statistical data analysis. *p* value < 0.05 was regarded as statistically significant. The *χ* ^2^ or Fisher exact tests, as well as Pearson's correlation were used to evaluate the association between gene expressions.

## 3 RESULTS

### 3.1 Downregulation of *MEIS1* in ESCC cell line KYSE‐30

**Figure 1:**
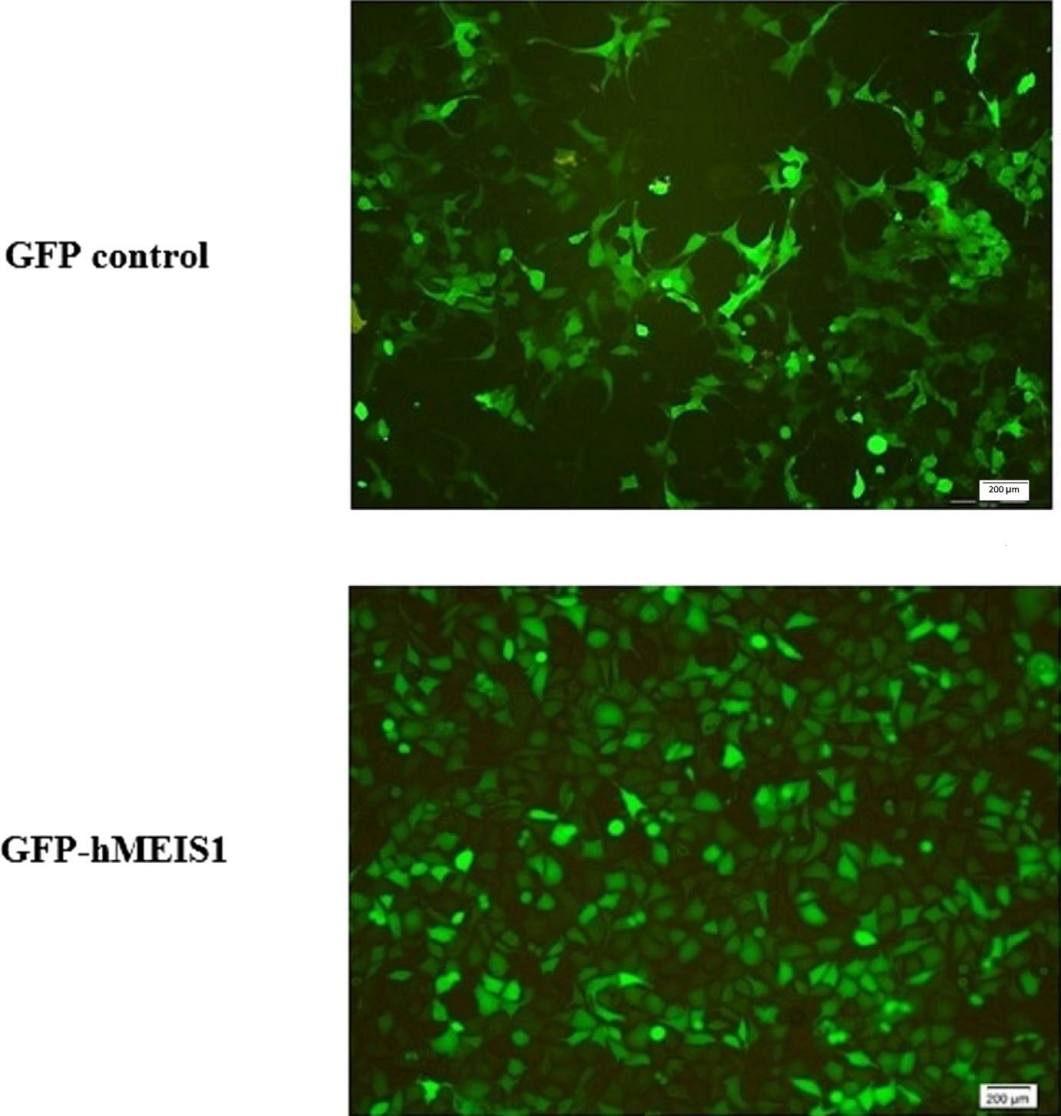
Knockdown of *MEIS1* gene expression in KYSE30 cells. Fluorescent microscopy images of GFP‐hMEIS1 and control cells 5 days after transduction

After lentiviral‐mediated *MEIS1* knockdown, the expression of *MEIS1* was evaluated in GFP‐hMEIS1 in comparison with pCDH513b GFP‐control KYSE‐30 transduced cells (>95% positive) to confirm *MEIS1* silencing. The fluorescent microscopy images of transduced GFP‐shMESI1 and GFP control KYSE30 cells are shown in Figure 1. The significant underexpression nearly 5.5 (log2 fold change) of *MEIS1* was detected in lentiviral GFP‐hMESI1 transduced cells compared to GFP control.

### 3.2 Knockdown of *MEIS1* changed the expression of differentiation genes

**Figure 2:**
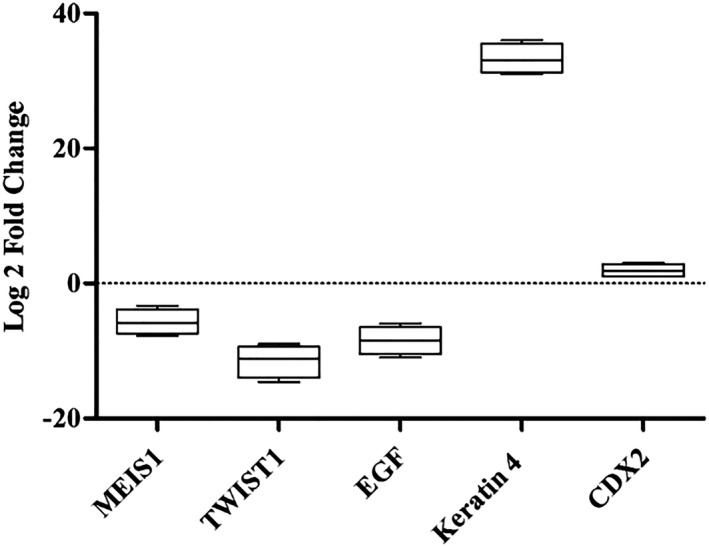
Knockdown of *MEIS1* gene has a significant impact on *TWIST1, EGF*, *KRT4*, and *CDX2* mRNA expression in KYSE‐30 cells. Retroviral transduction silenced *MEIS1* expression in GFP‐hTMEIS1 nearly 5.5‐fold compared to GFP control cells causing a – 12‐, ‐8‐fold decrease and 34‐, 1.14‐fold increase in *TWIST1, EGF, KRT4,* and *CDX2* mRNA expression, respectively

Having confirmed the *MEIS1* gene silencing in KYSE‐30 cells, we analyzed expression of specific epithelial and epithelial‐mesenchymal transition markers in examined cells. Downregulation of *MESI1* led to a significant decrease in the levels of *EGF* and *TWIST1* mRNA expression (‐8‐ and ‐12‐fold, respectively) in GFP‐hMESI1 compared to control cells. Additionally, *MEIS1* underexpression significantly increased expression of *KRT4* and *CDX2* mRNA levels with 34‐ and 1.14‐fold, respectively. The results are summarized in Figure 2.

## 4 DISCUSSION

Different biological processes are involved in tumorigenesis such as cell proliferation, apoptosis, differentiation, metastasis, vascularization, as well as self‐renewal. In addition, deregulation of signaling pathways can induce tumorigenesis and cancer progression. Accordingly, identification of the involved genes in tumor emergence and understanding of underlying molecular mechanisms are required for representing effective therapeutic targets (Forghanifard et al, 2014; Patel et al., 2016).

In this study, *MEIS1* was silenced in ESC cell line KYSE‐30 and its impact on different involved genes in cell differentiation was analyzed. Following *MEIS1* knockdown in KYSE‐30 cells, we revealed a significant increase in expression level of specific markers of differentiated epithelial cells including *KRT4* and *CDX2*, while a significant downregulation was observed in *EGF* and *TWIST1* gene expression, as specific markers of cell proliferation and EMT, respectively. These results may highlight the critical role of *MEIS1* in regulation of cell differentiation through modulating of gene expression pattern in ESC cell line KYSE‐30.

Homeobox genes function as master transcriptional regulator of stem cell (SC) differentiation from embryonic stages to adult tissues, and their aberrant expression is associated with tumorigenesis (Rodrigues, Esteves, Xavier, & Nunes, 2016). (Grier et al., 2005). Among homeobox genes, *MEIS1* involves in stem cell growth and self‐renewal, as well as cell development and differentiation during embryogenesis (Zhu et al., 2017). Moreover, it plays a critical role in tumorigenesis, and functions as a negative regulator of cell cycle checkpoints, as well as cell proliferation and apoptosis in some malignancies such as prostate, non‐small‐cell lung cancer (NSCLC), clear cell renal cell carcinoma (ccRCC), and ESCC (Aksoz et al., 2018; Crist et al., 2011).

Intriguingly, numerous reports have demonstrated that different molecular modulators are related to *MEIS1* expression including TFs, miRNAs, and cellular metabolites (Aksoz et al., 2018). In addition, *MEIS1* can modulate expression of involved genes in cell differentiation and proliferation such as *HIF1/2, GATA1, CCND1/3, SOX3,* and *PBX1* (Torres‐Flores, 2013). It has been confirmed that *MEIS1* is involved in progression of different malignancies. It functions as a double‐edged sword with a dual function (either oncogenic or tumor suppressive role) in diverse cell types depending on the cell context. Oncogenic role of *MEIS1* transcription factor is detected in a variety of malignancies including leukemia, malignant peripheral nerve sheath tumors (MPNST), nephroblastoma, and ovarian, where it promotes cell proliferation and inhibits programmed cell death (Blasi, Bruckmann, Penkov, & Dardaei, 2017). On the other hand, several studies have demonstrated that *MEIS1* acts as potential tumor suppressor in some tumors such as ccRCC, prostate, lung, gastric, and colorectal cancers through promoting cell differentiation and inhibition of epithelial cell proliferation (Chen et al., 2012; Song, Wang, & Wang, 2017; Zhu et al., 2017).

*MEIS1* silencing through RNAi mechanism was correlated with epithelial cancer cells (NSCLC) proliferation and accelerated cell cycle progression in vivo (Li, Huang, Guo, & Cui, 2014). Moreover, *MEIS1* knockdown inhibited DNA replication in acute lymphoblastic leukemias (ALL) through regulation of involved genes in cell cycle process (Orlovsky et al., 2011). On the other hand, *MEIS1* ectopic expression in gastric cancer (GC) cells not only suppressed critical cancer cell properties including cell proliferation, colony formation, anchorage independent growth, epithelial mesenchymal transition (EMT), migration, and invasion, but also induced apoptosis and cell cycle arrest at G1/S transition in vitro (Song et al., 2017). In addition, an inverse association between *MEIS1* and SRY (sex determining region Y)‐box 2 (*SOX2*) in ESCC tumor samples was reported (Rad et al., 2016). *MEIS1* silencing in ESC cell line KYSE‐30 has resulted in overexpression of *SOX2* as a stemness factor. Such results may proposed suppressive role of *MEIS1* on *SOX2* gene expression in ESCC to inhibit stemness state progression (Rad et al., 2016). It has been illustrated that *MEIS1* silencing in mouse embryonic carcinoma suppressed differentiation in neural cells, while its ectopic expression induced differentiation via expression of neural progenitor markers including *GLAST, BLBP, SOX1,* and Nestin (Yamada, Urano‐Tashiro, Tanaka, Akiyama, & Tashiro, 2013). Consequently, induced *OCT4* can increase *MEIS1* expression and the upregulated *MEIS1* can repress *OCT4* expression, as a main gene of pluripotency, in a negative feedback loop (Yamada et al., 2013). Thus, modulation of OCT4 and SOX2 protein expression occur via differentiation signals, and *MEIS1* is contributed in this modulation of tumor cell differentiation (Rad et al., 2016; Yamada et al., 2013).

During differentiation process, expression of differentiation markers is increased, while expression of Yamanaka factors (*OCT4, SOX2, KLF4,* and *MYC*), which are activated in embryonic stem cells, is decreased. In addition, high‐level expression of Yamanaka factors can alter the gene expression pattern of the cell from differentiated to de‐differentiated state that lead to the cell reprograming (Miyamoto, Furusawa, & Kaneko, 2015). Since tumor cells approximately exhibit markers and properties of embryonic stem cells, low level of *OCT4* or *SOX2* is necessity for supporting *MEIS1* expression to promote the maintenance of differentiation in such cells (Tucker et al., 2010).

Here we have sought to investigate the significant changes in expression level of some gene related to tumor cell differentiation following *MEIS1* silencing in ESC cell line KYSE‐30. *MEIS1* silencing resulted in suppression of the involved genes in cell proliferation (*EGF*) and EMT (*TWIST1*), leading to tumor cell differentiation in ESC cell line KYSE‐30.

TWIST1, as a bHLH transcription factor, is a key regulator of different cellular processes. It identify E‐box consensus sequence in promoter of target genes and adjust downstream gene expression (Izadpanah, Abbaszadegan, Fahim, & Forghanifard, 2017). It was revealed in this study that *MEIS1* knockdown causes a significant decrease in *TWIST1* gene expression in KYSE‐30 cells. *TWIST1* not only involves in embryonic organogenesis, specification, and differentiation, but also is associated with tumor initiation, angiogenesis, stemness and EMT (epithelial‐mesenchymal transition) promotion, leading tumor cell invasion and metastasis in a variety of human malignancies (Forghanifard, Rad, et al., 2017). It has been indicated that silencing of *TWIST1* lead to increase osteoblast differentiation in mesenchymal stem cells (MSCs) by upregulation of the involved genes in FGF/ERK and BMP signaling pathways (Miraoui, Severe, Vaudin, Pagès, & Marie, 2010). Upregulation of numerous Zn‐finger TFs such as *SNAIL1/2, ZEB1/2,* and *TWIST1/2* that involve in several cell signaling pathways can lead to loss of E‐cadherin, the hallmark of EMT progression (Cheng, Auersperg, & Leung, 2012; Forghanifard, Khales, et al., 2017). *TWIST1* ectopic expression leads to downregulation of E‐cadherin and activation of mesenchymal markers. Inverse correlation between upregulation of *TWIST1* and decreased expression of E‐cadherin has been shown in several malignancies (Sasaki et al., 2009). According to the role of *TWIST1* in EMT, its significant decreased expression following *MEIS1* silencing in KYSE‐30 cells may inhibit EMT progress and invasiveness behavior of the cells, and reverse the process of mesenchymal transition which may result in tumor cell differentiation.

*EGF* (epidermal growth factor), as a tyrosine kinase ligand, stimulates various cellular responses such as epithelial cells differentiation and proliferation, apoptosis, migration, as well as cell division and survival (Li, Shan, et al., 2014). Interaction between *EGF* and its receptor (EGFR or ErbB‐1) leads to activate growth factor‐mediated intracellular downstream pathways such as PI3K/AKT and RAF/MEK/ERK that result in *EGF*‐induced EMT (Bodnar, 2013). In this study, silencing of *MEIS1* expression significantly suppressed the *EGF* expression in KYSE‐30 cells indicating that *MEIS1* is involved in the *EGF* related signaling cascades. It is suggested that downregulation of *EGF,* as an epithelial factor, leads to decrease EMT (Li, Shan, et al., 2014). Taken together, our results illustrate that underexpression of *TWIST1* and *EGF*, as two prevalent TFs of EMT promotion, can lead to a suppressed EMT in KYSE‐30 cells as an invasive cell line.

We have found that stable *MEIS1* knockdown induces *KRT4* and *CDX2* upregulation, indicating these genes are involved in KYSE‐30 differentiation. *CDX2*, as a ParaHox family of homeobox genes, has a key functions in intestinal epithelial differentiation, proliferation, maintenance of the intestinal phenotype and regulation of intestine specific gene transcription program, WNT‐mediated beta‐catenin signaling as well as tumorigenesis (Dong & Guo, 2015). In addition, downregulation of *CDX2* leads to development of intestinal neoplasia and is introduced as a prognostic marker for colon cancer (Dong & Guo, 2015). Ectopic expression of the intestine‐specific homeobox transcription factor *CDX2* cause Barrett's esophagus and gastric‐intestinal metaplasia (Joo, Park, & Chun, 2016). Furthermore, loss of *CDX2* expression was found in various ESC cell lines due to promoter hypermethylation (Guo et al., 2007). In line with these reports, our results also confirmed this pattern of the gene expression. While the EMT involving genes were downregulated and EMT process was suppressed, the epithelial markers were upregulated to fix epithelial state of the cells inhibiting mesenchymal converting. Since all these changes in gene expression pattern was induced by *MEIS1* knockdown, it may be hypothesized that *MEIS1* promotes EMT and suppresses cell differentiation in ESC cell line KYSE‐30.

Cytokeratins (CKs), as intermediate filament cytoskeletal proteins, are the major components of normal epithelium and squamous tumor tissues which are expressed in different grades of cell differentiation and introduced as indicator for predicting of tumor progression in ESCC (Cintorino et al., 2001; Singh et al., 2009). *KRT4,* as a member of intermediate filament proteins family, is expressed in suprabasal layers of nonkeratinizing stratified epithelium such as esophagus and regulated in a differentiation‐dependent manner (Alam, Sehgal, Kundu, Dalal, & Vaidya, 2011). Interestingly, expression level of KRT4 protein was decreased in transition from normal esophageal epithelium to invasive tumor of stratified squamous epithelium and associated with cancer progression (Chung et al., 2006). Therefore, the increased expression of *KRT4* after *MEIS1* silencing may suggest the putative correlation of *KRT4* overexpression with differentiation state of the KYSE‐30 cells. Following stable *MEIS1* gene silencing, we have found a significant increase in *CDX2* and *KRT4* mRNA expression in KYSE‐30 cells, which may probably orient the cells toward differentiation phenotype. Altogether, these results correlate *MEIS1* expression and the involved genes in maintenance of tumor cell differentiation, introducing *MEIS1* as a probable key regulator in this process in ESC cells, and presenting a potentially molecular mechanism for regulation differentiation and EMT processes in ESC cell line KYSE‐30.

In conclusion, we showed that *MEIS1* is significantly correlated with the involved genes in cell differentiation and EMT processes in KYSE‐30 cells. Having confirmed the correlation of *MEIS1* with *TWIST1* and *EGF*, as well as its inverse association with epithelial cell markers including *KRT4* and *CDX2*, we may propose a role for *MEIS1* in progress of KYSE‐30 cell dedifferentiation. These findings may suggest that *MEIS1* gene repression can be a therapeutic strategy to inverse invasive characteristic of the ESC cells. To the best of our knowledge, this is the first report revealing regulatory role of *MEIS1* on expression of the involved genes in EMT and differentiation in esophageal squamous carcinoma cell line KYSE‐30.

## CONFLICT OF INTEREST

The authors declare no conflicts of interest.
